# Dll4-Notch signaling as a therapeutic target in tumor angiogenesis

**DOI:** 10.1186/2045-824X-3-20

**Published:** 2011-09-18

**Authors:** Frank Kuhnert, Jessica R Kirshner, Gavin Thurston

**Affiliations:** 1 Regeneron Pharmaceuticals, 777 Old Saw Mill River Road, Tarrytown, NY 10591, USA

## Abstract

Tumor angiogenesis is an important target for cancer therapy, with most current therapies designed to block the VEGF signaling pathway. However, clinical resistance to anti-VEGF therapy highlights the need for targeting additional tumor angiogenesis signaling pathways. The endothelial Notch ligand Dll4 (delta-like 4) has recently emerged as a critical regulator of tumor angiogenesis and thus as a promising new therapeutic anti-angiogenesis target. Blockade of Dll4-Notch signaling in tumors results in excessive, non-productive angiogenesis with resultant inhibitory effects on tumor growth, even in some tumors that are resistant to anti-VEGF therapies. As Dll4 inhibitors are entering clinical cancer trials, this review aims to provide current perspectives on the function of the Dll4-Notch signaling axis during tumor angiogenesis and as a target for anti-angiogenic cancer therapy.

## Introduction

The concept that solid tumors require the growth of new blood vessels (angiogenesis) for oxygen and nutrient supply, first proposed by Folkman 40 years ago [1], is now solidly accepted and has spurred substantial efforts to develop anticancer therapeutics that interfere with tumor angiogenesis. Many of the angiogenic signaling pathways necessary for embryonic development are reactivated during tumor angiogenesis, and as such represent targets for anti-angiogenic cancer therapy. VEGF (vascular endothelial growth factor) is a primary endothelial cell growth factor, and blockade of the VEGF signaling pathway is now a clinically approved and widely used therapy for cancer. However, inherent or acquired resistance to anti-VEGF therapy is frequently observed in tumors, thus illustrating the need for targeting additional angiogenesis pathways to fully exploit the promise of anti-angiogenic cancer therapy. Notch signaling has recently emerged as a critical regulator of developmental and tumor angiogenesis. Notch signaling in both endothelial and smooth muscle cells appears to provide critical regulatory information to these cells downstream of the initiating signal induced by VEGF. In particular, the Notch ligand Dll4 (delta-like 4) has been identified as a promising new target in tumor angiogenesis in preclinical studies. Pharmacological Dll4 inhibitors have been developed and are entering clinical trials for solid tumors. This review aims to provide current perspectives on the function of Dll4-Notch signaling axis during tumor angiogenesis and on mechanisms and applications of targeting this pathway for cancer therapy.

### The Delta/Jagged-Notch signaling pathway

The Notch pathway is an evolutionary conserved signaling system that regulates cell fate specification, tissue patterning and morphogenesis by modulating cell differentiation, proliferation, apoptosis and survival [2-4]. In mammals, the core components of the pathway include five canonical DSL (Delta, Serrate, Lag2) ligands (called Dll1, 3, 4, and Jagged1 and 2) and four single-pass transmembrane receptors (Notch1-4). Since the DSL ligands are membrane-bound, the Notch pathway relies on direct cell-cell interactions for signal generation. Ligand binding to the extracellular domain of Notch triggers the proteolytic activation of the receptor. Juxtamembrane region cleavage of Notch by ADAM metalloproteinase is followed by γ-secretase complex-mediated cleavage and generation of the Notch intracellular domain (NICD). NICD then translocates to the nucleus, where it interacts with the RBPJ/CSL transcription factor and induces the expression of Notch target genes such as the basic helix-loop-helix proteins Hes and Hey.

### Dll4-Notch signaling in vascular development

Functional studies in mice, zebrafish and cultured endothelial cells have demonstrated a critical role for Notch signaling during formation of the vascular system (for recent comprehensive reviews see [5-7]). Of the four Notch receptors, Notch1 and Notch4 are expressed by endothelial cells [8]. Gene targeting studies in mice have demonstrated that Notch1 is the primary functional Notch receptor during developmental angiogenesis [9]. Except for Dll3, expression of all Notch ligands has been detected in endothelial cells [5]. Dll4 is the first Notch ligand to be expressed during mouse development, and Dll4 transcripts were detected in most capillary beds and arterial vessels [10,11]. Lack of a single Dll4 allele in mice leads to early embryonic lethality characterized by severe defects in arterial differentiation and vascular remodeling [12-14].

A clearer picture of Dll4 function during vascular morphogenesis has emerged from subsequent studies demonstrating that one function of Dll4 is to regulate the specification of endothelial cells into tip and stalk cells during angiogenic sprouting [15-20]. Dll4 is induced in endothelial tip cells of angiogenic sprouts in response to VEGF [15,17,21] and activates Notch in adjacent stalk cells. Mosaic analysis has demonstrated that Notch is required cell-autonomously for stalk cell specification by actively repressing tip cell phenotypes [15]. Loss of Dll4 expression leads to dramatically increased capillary sprouting and branching as a result of excessive tip cell formation and endothelial proliferation. Thus Dll4-Notch signaling functions as a regulator of angiogenesis downstream of VEGF. The loss of Notch signaling is associated with an increase in VEGF receptor (VEGFR)-2 and VEGFR-3 expression in stalk cells [20,22,23], indicating that Notch can provide negative feedback to reduce the activity of the VEGF/VEGFR axis.

### Dll4-Notch signaling in tumor angiogenesis

Studies in humans and mice have demonstrated that Dll4 is strongly expressed by the tumor vasculature and generally not by the tumor cells themselves. In various mouse models, strong Dll4 expression was observed in the majority of tumor vessels, contrasting with significantly lower vascular expression in adjacent normal tissues [11,13,24]. Paralleling vascular development, Dll4 expression in tumor vessels appears to be directly regulated by VEGF; thus Dll4 expression levels in tumors have been found to correlate with those of VEGF [24,25]. In humans, Dll4 expression was analyzed in tumors from kidney, bladder, colon, brain and breast [11,25-29]. Robust Dll4 expression was observed specifically in the tumor vasculature in all of these tumor types, whereas Dll4 expression was low to undetectable in the vasculature of adjacent normal tissue. For example, Dll4 expression in renal clear cell tumors was confined to the vasculature and detected at nine-fold higher levels than in normal kidney [11,29]. Dll4 expression was generally not observed in the tumor parenchyma, although sporadic tumor cell expression was detected in colorectal and brain tumor samples [27,28]. Interestingly, although most colorectal and breast tumors showed positive Dll4 expression in tumor vessels, some tumors were negative. Further, at least in the case of breast cancer, the degree of Dll4 expression correlated with outcome: tumors with high Dll4 in the vasculature progressed more rapidly [26]. More work is needed to understand the factors that regulate Dll4 expression in tumors and to extend the connection between Dll4 expression and tumor progression.

Consistent with its role in endothelial tip/stalk cell specification during development, inhibition of Dll4-Notch signaling caused increased vascular density and vascular sprouting in tumors [24,28,30,31]. Surprisingly, this vascular overgrowth phenotype resulted in tumor growth inhibition in a variety of human and rodent tumor models [24,30,31]. Perfusion studies demonstrated that the hypersprouting tumor vasculature was non-functional and consequently, anti-Dll4-treated tumors exhibited increased levels of hypoxia. Thus, blockade of Dll4-Notch signaling leads to tumor vessel "abnormalization" (i.e. the formation of a hypersprouting, non-functional vasculature) with resultant growth inhibitory effects on tumors [32].

Gene targeting studies suggest that Notch1 is the primary Notch receptor during developmental angiogenesis [9]. Therefore it appears plausible that Notch1 is also the predominant mediator of Dll4-Notch signaling in the tumor vasculature, although Notch4 has also been described as a receptor specifically expressed by endothelial cells. Recently generated Notch1-specific inhibitory antibodies exhibited tumor vessel effects similar to those seen following blockade to Dll4 [33]. Additional Notch receptor-specific reagents or new conditional genetic mouse models will be instrumental for delineating the relative contribution of Notch1 and Notch4 to tumor angiogenesis.

Notch ligands other than Dll4 may also affect tumor angiogenesis. For instance, Jagged1 expression was detected in clinical breast cancer samples [34,35], while pro-angiogenic, paracrine functions for Jagged1 in head and neck squamous cell carcinoma have also been suggested [36]. Similarly, Jagged1 tumor angiogenesis-promoting effects were inferred in a mouse mammary tumor model [37]. Of note in this context, opposing effects of Dll4 and Jagged1 on endothelial sprouting were recently reported in the retina angiogenesis model [38]. Finally, Dll1 was implicated in the regulation of tumor angiogenesis in a fibroblast tumor model [39].

### Mechanisms of Dll4-Notch inhibition on tumor vessels

**Figure 1:**
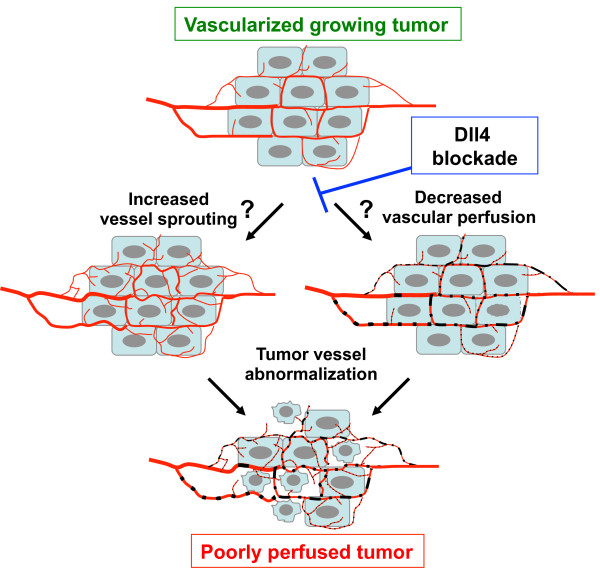
**Effects of Dll4-Notch inhibition on tumor angiogenesis**. Blockade of Dll4-Notch signaling in endothelial cells leads to tumor vessel abnormalization characterized by increased tumor vessel sprouting and decreased vascular perfusion. It is currently not clear whether hypersprouting leads to decreased tumor perfusion (left hand axis) or decreased perfusion leads to hypersprouting (right hand axis), or whether both occur in parallel. The overall effect of Dll4-Notch blockade is decreased tumor perfusion and reduced tumor growth.

Blockade of Dll4-Notch resulted in tumor growth inhibition in a variety of human and rodent tumor models associated with the formation of a non-functional, hypersprouting tumor vasculature [24,30-32]. Perfusion studies demonstrated that the hypersprouting tumor vasculature was non-functional, rendering the tumors more hypoxic (tumor vessel abnormalization) [32]. However the precise mechanisms underlying the rapid reduction of tumor perfusion following Dll4-Notch inhibition are not fully understood. Indeed, it is not known whether endothelial cell hypersprouting can lead to reduced tumor perfusion and is thus a primary upstream event, or whether an initial loss of tumor vessel perfusion leads to hypersprouting (Figure 1).

In one set of studies, reduced pericyte coverage and increased vascular leakage were observed in tumors treated with Dll4-Notch inhibitors [40,41]. Such an increase of vascular leakiness associated with impaired vascular integrity may explain a rapid decrease in tumor perfusion. Another possibility is that the abnormalized network as a whole loses the organization or vaso-regulation necessary to support adequate perfusion [30,32].

Alternatively, it is plausible that initiation of hypersprouting structures that are devoid of lumens would result in a loss of perfusion. Endothelial lumen formation is an essential step during vascular morphogenesis, and several mechanisms have been described for lumen formation in different vascular beds, including the coalescence of vacuoles within endothelial cells and the apical surface specialization between adjacent endothelial cells [42,43]. Tumor vessel obstruction by intraluminal endothelial cells was recently reported in response to γ-secretase inhibitor treatment in both an orthotopic renal cell carcinoma and a VEGF-driven rabbit hind limb angiogenesis model [41]. In contrast, however, a recent study demonstrated reduced microvascular occlusion and the modulation of vasoconstriction following inhibition of Dll4-Notch signaling during post-angiogenic remodeling in retinal angiogenesis [44].

While it is suggested that endothelial hypersprouting may lead to disorganized cellular organization and ultimately vessel obstruction, this phenomenon is also reminiscent of the loss of cell polarity phenotype recently reported in endothelial β1 integrin mutants [45]. Endothelial cell polarization, linked to the specialized apical-basal distribution of cell adhesion molecules and their interaction with the Par polarity complex [42,43], is a necessary step for the formation of a patent vascular lumen. For example, loss of endothelial β1 integrin results in the loss of Par3 expression and the mislocalization of cell adhesion proteins Claudin-5, PECAM-1, VE-cadherin and CD99 specifically in arteries [45]. The activation of β1 integrin by Dll4-Notch1 in a CSL-independent manner [46] suggests a possible link between Notch signaling and endothelial cell polarity.

CCM1 is another emerging regulator of endothelial cell polarization and lumen formation [47]. CCM1 interacts with VE-cadherin and directs adherens junction organization, distribution and association with the Par polarity complex in cultured endothelial cells and in human cerebral cavernous malformation (CCM) lesions [47]. CCM1 interacts with the intracellular protein ICAP1, which in turn binds specifically to β1 integrins [48]. Both CCM1 and ICAP regulate endothelial cell quiescence and angiogenic sprouting by activating Dll4-Notch signaling [49]. Thus, it is possible that some of the cell polarizing functions of CCM1 are mediated by Notch signaling. Based upon these associations, further analysis of the expression and distribution of polarity markers in the context of Dll4-Notch inhibition may provide additional evidence for a role for Notch signaling in regulating lumen formation and/or maintenance.

### Interaction of the Dll4-Notch axis with other signaling pathways

**Figure 2:**
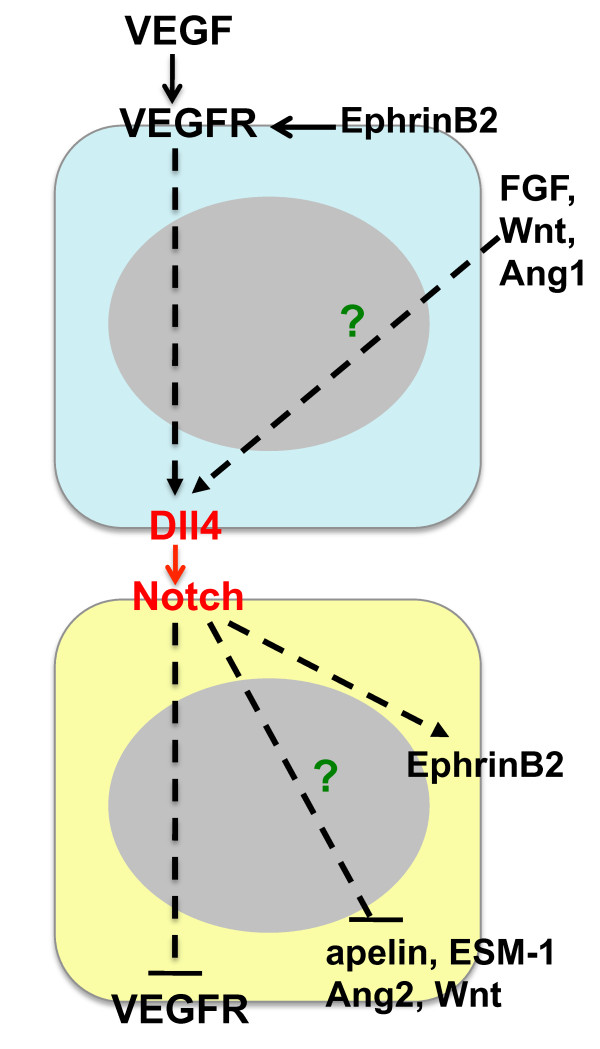
**Interaction of the Delta-Notch axis with other signaling pathways in endothelial cells**. Solid lines denote protein-protein interactions, while dashed lines indicate transcriptional regulation. Binding of VEGF to VEGF receptors (VEGFR2) results in the transcriptional up-regulation of Dll4. Dll4-mediated activation of the Notch receptor on an adjacent endothelial cell leads to the transcriptional repression of VEGFR expression. EphrinB2 is a Dll4-Notch target gene, but also acts upstream of Dll4-Notch signaling as a regulator of VEGFR endocytosis and signaling. Other putative signaling pathways downstream of Dll4-Notch include apelin, ESM-1, Ang2 and Wnt, while FGF, Wnt and Ang1 are potential upstream activators of Dll4 expression.

Studies in various model systems have established that the VEGF pathway can regulate the expression of Notch signaling components [29,50]. Up-regulation of Dll4 expression by VEGF has been demonstrated in cultured endothelial cells and in endothelial tip cells in the retina angiogenesis model [17,20,29,30,51]. Similarly, strong expression of Dll4 on the growing front of tumor vessels and VEGF regulation of Dll4 in tumors has been documented [24]. Conversely, down-regulation of VEGFR2 and VEGFR3 expression following Notch activation was found in cultured endothelial cells and endothelial stalk cells [20,22,52]. Down-regulation of VEGFR2 and/or VEGFR3 has been proposed as a mechanism to reduce endothelial proliferation and migration and to permit local differentiation of cells within a zone of VEGF-driven angiogenesis [7]. These finding suggest that Notch can provide negative feedback to reduce the activity of the VEGF/VEGFR2/VEGF3 axis during angiogenic sprouting (Figure 2). The emerging picture is that VEGF acts as a central driver of angiogenesis, while Notch signaling helps to coordinate the response appropriately [53].

Although this model also applies to tumor angiogenesis, additional complexities appear to exist in the tumor microenvironment [50]. For instance, the fact that potent combination effects are observed for the simultaneous blockade of Dll4 and VEGF [30] is difficult to reconcile with a simple linear negative feedback model. Pathways other than VEGF, such as FGF, Angiopoietin-1/Tie2, Wnt, inflammatory cytokines, extracellular matrix components or Notch signaling itself were also shown to induce the expression of Dll4 in endothelial cells. Thus, it is plausible that some of these additional factors also act upstream of Dll4 in the tumor environment [30,54-58] (Figure 2).

EphrinB2 has been identified as key downstream target gene of Dll4-Notch in cultured endothelial cells and during endothelial specification towards the arterial fate in mouse and zebrafish [6]. More recently, EphrinB2 regulation of VEGFR2 endocytosis and signaling in endothelial tip cells upstream of Dll4-Notch was reported [59,60] (Figure 2). Targeted disruption of EphrinB2 results in early embryonic lethality due to angiogenic remodeling defects [61,62]. It is of great interest to determine how EphrinB2 affects the tumor vasculature in the presence of high levels of VEGF. Suppression of EphrinB2 signaling using soluble EphrinB2-Fc in a subcutaneous squamous cell carcinoma model phenocopied the effects of Dll4 blockade, in particular, reduced tumor growth accompanied by the induction of non-productive angiogenesis [63], suggesting that EphrinB2 acts as a downstream mediator of Dll4/Notch function. Conversely, targeted inactivation of EphrinB2 PDZ-dependent reverse signaling led to decreased vascularization and reduced endothelial sprouting, that is, the normalization of the tumor vasculature, in an orthotopic glioma model [59]. Similarly, blockade of EphrinB2 signaling with soluble EphB4-Albumin resulted in reduced tumor vascularization in the RIP1-Tag2 model of pancreatic islet carcinogenesis [40], consistent with an inhibitory activity on VEGF signaling and a function upstream of Dll4. It is possible that EphrinB2 inhibition exhibits specific effects depending on the tumor model or reagent. Further studies are warranted to elucidate EphrinB2 function during tumor angiogenesis, particularly in the context of anti-VEGF and/or anti-Dll4 combination therapies.

Several tip cell-enriched genes have recently been identified in the mouse retina which may yield clues to which signaling pathways mediate the effects of Dll4-Notch blockade on the tumor vasculature [64,65]. Of particular interest are the secreted molecules ESM-1, angiopoietin-2 and apelin. Binding of these factors to their cognate receptors on stalk cells and the regulation of retinal angiogenesis and endothelial proliferation by the apelin/APJ signaling axis were demonstrated [64]. Additionally the induction of Wnt signaling activity in endothelial cells via the Notch target gene Nrarp was recently shown [66]. The functional significance of these pathways in the context of Dll4-Notch blockade in tumors remains to be determined.

### Therapeutic Inhibition of Dll4-Notch signaling during tumor angiogenesis

Pharmacological targeting of Dll4-Notch signaling in preclinical tumor models has been achieved by several different mechanisms including anti-Dll4 antibodies, DNA vaccination, soluble Dll4-Fc and Notch-Fc decoys, Notch antibodies, and γ-secretase inhibitors [24,28,30,31,37,41,67,68]. Unlike γ-secretase inhibitors that broadly block all Notch signaling, specific targeting of Dll4 with anti-Dll4 antibodies did not induce overt gastrointestinal toxicity and has thus emerged as an attractive target for anti-angiogenic cancer therapy [30,69]. Consequently, anti-Dll4 antibodies have recently entered clinical trials for the treatment of advanced solid malignancies.

A clinically important question is what types of cancer would benefit most from ani-Dll4 therapy. Thus far, tumor vascular Dll4 expression has been detected in many human tumor samples including kidney, bladder, colon, brain and breast cancer [11,25-29]. Predictive biomarkers produced by the tumor or expressed within the tumor vessels that confer sensitivity to Dll4-Notch blockade have not yet been identified. A useful starting point may be to assume that high levels of Dll4-Notch are correlated with sensitivity to pathway inhibition [50]. Thus, further analysis of the expression levels of various components of the Delta-Notch signaling pathway in clinical specimens will be useful.

Inhibition of VEGF signaling was the first clinically approved therapy targeting angiogenesis in cancer. Anti-VEGF therapy has widespread activity against multiple tumor types, but the effects are variable and incremental, and acquired or innate resistance is frequently encountered [70,71]. Anti-VEGF therapy acts to prune vascular sprouts and reduce new vessel growth [32,72], in contrast to the cellular effects of blocking Dll4-Notch described above. Importantly, preclinical studies have shown that blockade of Dll4 can have potent growth inhibitory effects on tumors that are resistant to anti-VEGF therapies [24,30]. Furthermore, the simultaneous targeting of Dll4 and VEGF has produced additive anti-tumor effects compared to single agents in a number of tumor models ([30]; Kirshner and Thurston, unpublished). These observations clearly raise the possibility of combining anti-angiogenic therapies against these two pathways. Although blocking one pathway might sensitize tumor vessels to inhibition of the other, much remains to be learned at the mechanistic levels as to how these two pathways interact during tumor vascularization; certainly not all of the above findings can be explained by a simple linear VEGF-Dll4-Notch4 feedback loop as described in the retinal angiogenesis model [20,22].

The preclinical evaluation of combining Dll4 inhibition and cytotoxic agents represents another area of great clinical importance. Blockade of VEGF exhibits clinical potency predominantly when combined with chemo- or radiation therapy. Preclinical studies have suggested a model in which the normalization of tumor vessels achieved by anti-VEGF therapy allows for more efficient delivery of oxygen and drug into the tumor [73]. Although blockade of Dll4 results in an abnormalization of the tumor vasculature, combining Dll4 inhibition with cytotoxic chemotherapy frequently results in enhanced anti-tumor activity in preclinical tumor models ([68]; Kirshner and Thurston, unpublished). In addition to the anti-angiogenic mechanism of action of disrupting Dll4 in the tumor vasculature, direct effects on the frequency of tumor initiating cells (cancer stem cells) and tumor growth have been described for tumor cell-specific targeting of Dll4 alone or in combination with chemotherapy [68]. Additional studies are warranted to elucidate the mechanism of this combination therapy approach and to ascertain the clinical validity of this treatment option.

### Effects of Dll4-Notch blockade on normal organs

Dll4 is strongly upregulated in tumor endothelium compared to normal organs, however it is expressed to some degree in smaller arteries and capillaries of normal tissues, as well as in the thymic stroma and the gastrointestinal tract [10,13,25-27]. The differential expression between tumor and normal vessels likely explains the preferential targeting of Dll4-expressing tumor endothelial cells in preclinical tumor models. However, clinical development of Dll4 inhibitors requires careful evaluation of possible adverse effects to establish a formal therapeutic index.

In addition to a role in tumor angiogenesis, Dll4-Notch signaling also plays a crucial role in T-cell lymphocyte development and differentiation. Gene targeting studies have demonstrated that Notch1 is the essential Notch receptor for T-cell lineage commitment, while Dll4 is the Notch ligand required to induce Notch signaling in thymic immigrant cells and to actively determine T-cell fate [74-76]. Dll4 is constitutively expressed on the thymic epithelial cells (TEC) as well as on endothelial cells [13,24]. Specific deletion of Dll4 in thymic epithelial cells throughout development results in a complete block of T-cell development, associated with thymic acellularity and ectopic appearance of immature B cells [75,76]. Subsequent studies using pharmacologic blockade of Dll4 demonstrated that ongoing Dll4-Notch signaling is required for T-cell differentiation in the adult murine thymus [77]. These latter studies also demonstrated the reversibility of the thymus/T-cell phenotype upon cessation of treatment with anti-Dll4 antibody, which has important implications for potential clinical use of Dll4-Notch inhibitors.

A recent report demonstrated that chronic Dll4 blockade induced vascular proliferation in liver of mice, rats, and monkeys with associated hepatocellular changes, as well as the development of subcutaneous vascular lesions in rats, referred to as neoplasia [78,79]. In genetic mouse models, loss of Dll4 or Notch1 function also led to the activation of liver endothelial cells and the formation of hepatic vascular lesions [40,80], although lesions in the skin were not reported. Of note, vascular lesions in liver induced by anti-Dll4 antibody administration appeared to be highly dose-dependent [78,79]. The most pronounced effects were observed at the highest doses, which may be beyond what is needed for blocking Dll4 in tumor vessels in clinical settings. Further, the effects on liver function were shown to be reversible following cessation of treatment [79]. It will be important to assess whether similar pathological changes are observed with doses of anti-Dll4 antibody that are clinically relevant.

In one study, the hepatic vascular alterations observed in the Dll4 loss of function mice could be prevented by concomitant treatment with the EphrinB2 signaling inhibitor sEphB4-Alb. Interestingly, the simultaneous blockade of these signaling pathways displayed additive inhibitory effects on pancreatic tumor growth and perfusion [40]. The role of EphrinB2 in this study is consistent with its recently described function as an inhibitor of VEGF signaling in endothelial tip cells upstream of Dll4-Notch [59,60]. This raises the intriguing possibility that the induction of vascular changes in some organs can be manipulated by combination anti-angiogenesis therapy, while at the same time additive anti-tumor effects are achieved.

### Concluding Remarks and Future Directions

The endothelial cell Notch ligand Dll4 has recently emerged as a critical regulator and a promising target in tumor angiogenesis. Blockade of Dll4-Notch signaling in tumors results in excessive, non-productive angiogenesis and inhibitory effects on tumor growth. Thus inhibition of Dll4 functions by a very different anti-angiogenic mechanism than therapies targeting the VEGF pathway. Significantly, enhanced anti-tumor effects in preclinical models have been observed by combined inhibition of Dll4 and VEGF, and blockade of Dll4 was found to have potent growth inhibitory effects on some tumors that are resistant to VEGF inhibition.

From a mechanistic perspective, several important questions require further elucidation. Specifically, the mechanisms underlying the reduced tumor vascular perfusion induced by Dll4-Notch inhibition remain unclear. For example, what are the primary upstream events that lead to reduced tumor perfusion, and what are the effects on endothelial cell polarization and lumen formation? The characterization of signaling pathways other than VEGF that either act upstream to regulate Dll4 expression or downstream to mediate the effects of Dll4-Notch signaling also warrants further study. From a therapeutic standpoint, it will be instrumental to identify the tumor types that will benefit most from ani-Dll4 therapy and to further validate combination approaches with anti-angiogenic and chemotherapeutic regimens. Additionally, the careful evaluation of adverse effects on normal organ homeostasis for therapeutically-relevant doses of Dll4 inhibitors will be critical for advancement of Dll4 blocking agents in the clinic.

## Competing interests

The authors declare that they have no competing interests.

## Authors' contributions

FK and GT wrote the manuscript. FK, JRK and GT contributed conceptual information and edited the manuscript. All authors read and approved the final manuscript.
